# Towards reliable IoT communication and robust security: investigating trusted schemes in the internet of medical things using blockchain

**DOI:** 10.1038/s41598-023-47989-7

**Published:** 2023-11-24

**Authors:** Geetanjali Rathee, Rajagopal Maheswar, Sountharrajan Sehar, Durga Prasad Bavirisetti

**Affiliations:** 1 Department of Computer Science and Engineering, Netaji Subhas University of Technology, Dwarka Sector-3, New Delhi-110078, India; 2 Department of Applied Cybernetics, Faculty of Science, University of Hradec Kralove, Hradec Kralove, Czech Republic; 3 Department of Computer Science and Engineering, Amrita School of Computing, Chennai, Amrita Vishwa Vidyapeetham, India; 4 Department of Computer Science, Norwegian University of Science and Technology, Trondheim, Norway

**Keywords:** Outcomes research, Engineering

## Abstract

The Internet of Things (IoT) is evolving in various sectors such as industries, healthcare, smart homes, and societies. Billions and trillions of IoT devices are used in e-health systems, known as the Internet of Medical Things (IoMT), to improve communication processes in the network. Scientists and researchers have proposed various methods and schemes to ensure automatic monitoring, communication, diagnosis, and even operating on patients at a distance. Several researchers have proposed security schemes and approaches to identify the legitimacy of intelligent systems involved in maintaining records in the network. However, existing schemes have their own performance issues, including delay, storage efficiency, costs, and others. This paper proposes trusted schemes that combine mean and subjective logic aggregation methods to compute the trust of each communicating device in the network. Additionally, the network maintains a blockchain of legitimate devices to oversee the trusted devices in the network. The proposed mechanism is further verified and analyzed using various security metrics, such as reliability, trust, delay, beliefs, and disbeliefs, in comparison to existing schemes.

## Introduction

The emergence of smart technologies in the recent years has improved the quality and availability of services in various applications. The Internet-of-Things is evolving in various sectors such as industries, healthcare, smart homes, societies etc.^1^. In order to improve the efficiency and communication process in the network. Amongst various application, e-heath systems are considered as one of trending and latest interest of users for improving the health activities after the pandemic.

**Figure 1:**
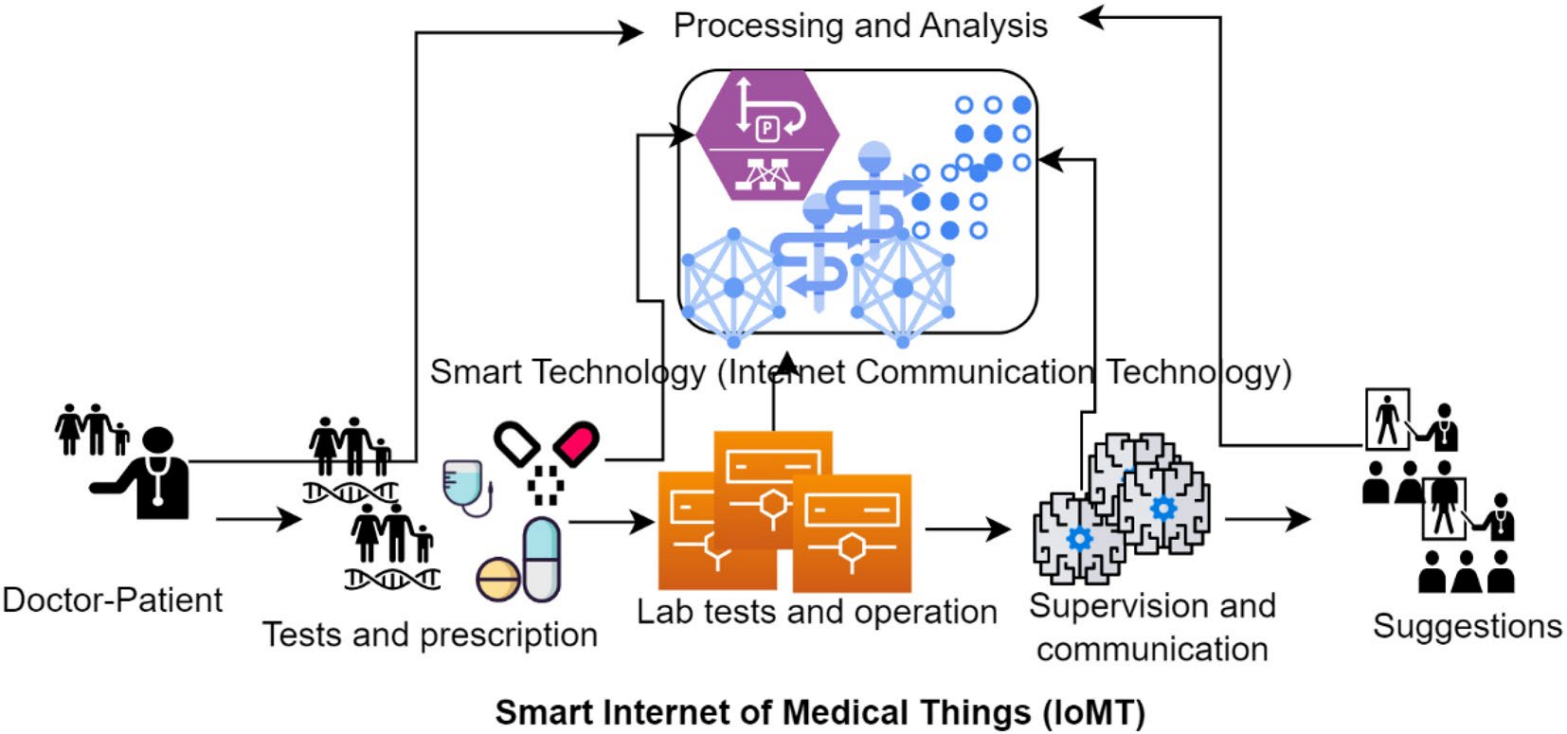
Remote heath monitoring framework using intelligent devices^6^.

Billions and trillions of IoT devices are used in e-health systems known as Internet of Medical Things (IoMT) for improving the communication process in the network. Scientists/researchers have proposed various methods and schemes to ensure automatic monitoring, communication, diagnosis and even operating the patience at a distance^2,3^. The e-health system not only reduces the gap among doctor and patient where they can coordinate or communicate without waiting in long queues and to be physically present in the hospital^4^. The e-health system also improves the operation and diagnosis along with management of patient medical records in a more efficient and effective way. The use of communication and information techniques in healthcare sectors has significantly improved the development and efficient utilization of resources of health records. The modern e-health systems are improving the functionality and availability of resources such as persons and monitoring conditions in good ways in various developing countries^5^. Figure 1 depicts the smart healthcare monitoring system where the management of records and communication among patient and doctor can be easily managed and examined in a more efficient, automatic and good way^6^. In case where a patient wants to change his/her doctor, then instead of repeating the diagnosis, tests and medicines the entire history of patient is automatically transferred to the other doctor whether it a part of same hospital or in any other hospital. The smart devices in healthcare system helps of automating the things via online meetings, record management and diagnose of patient using intelligent systems.

### Motivation

The e-healthcare systems where the monitoring or recording of patient’s information can be managed electronically or intelligent systems. The in-between entities that are responsible to manage the records may easily benefit the opportunity simply by stealing the medical records by selling it to this party. The third party may be life insurance companies or stakeholders where intermediators may steal the record where they can contact with the patient for insurances of researchers that can further use the data for their analysis. This will not only breach the patient’s secrecy but also benefit the health systems in their own way. There exist number of benefits and advantages of using intelligent devices while interacting or maintaining the records, however, the automation mechanism where doctors or hospitals can fully rely on smart devices for the final decisions or maintenance of records are not completely adopted by the organizations. The number of security factors while storing of patient’s medical records can be easily erased or altered by compromising the devices^7,8^.

Several researchers/scientists/authors have proposed security schemes and approaches for identifying the legitimacy of intelligent system that are involved in maintain the records in network. Though, the existing schemes have their own performance issues such as delay, storage efficiency, costs and other. Therefore, it is still needed to propose an efficient and trusted health system that can remotely managed with security and efficiency by the system.

### Contribution

The objective of this paper is to propose an integration of blockchain and trusted scheme for maintaining the records and ensuring transparency in the system. The trusted schemes are the combination of subjective logic and mean aggregation methods that computes the trust of each communicating device in the network. The trusted schemes in comparison of any other cryptographic, algorithmic method is much more efficient by reducing the extra storage and delay in the network. The trust of each device is computed in the network that can directly be used to identifying the legitimacy of the device. The device having higher trust rate are defined as the most trusted devices that are always elaborated in the communication process^9^. In addition, the blockchain of legitimate devices are maintained by the network to keep surveillance the trusted devices in the network. The proposed mechanism is further verified and analyzed using various security metrices in comparison of existing schemes such as reliability, trust, delay and beliefs and disbeliefs. The contribution of the paper is defined as follows:

- A subjective logic and mean aggregate schemes are integrated to strengthen the trust computation process for identifying the legitimacy of each communicating device in the network.
- A blockchain is maintained having trusted communicated devices for continuous surveillance where in case of future alteration of devices can be permanently removed from the network.
- The proposed mechanism is verified and validated against security metrics such as reliability, trust, delay and beliefs and disbeliefs.

The remaining behavior of the paper is organized as follows. Section “Related work” illustrates the number of various security schemes and approaches proposed by several scientists/researchers. A secure and transparent communication mechanism for remote health system is discussed in detail in Sect. “Proposed method”. Further, the analysis and verification of proposed mechanism is deliberated in Sect. “Performance analysis”. Finally, Sect. “Conclusion” concludes the paper along with future directions for improving the present work.

## Related work

This section deliberates several security schemes or approaches proposed by various researchers/scientists/authors for maintaining a secure, efficient and remote health system using various cryptographic, algorithmic and computational approaches. Table 1 describes the number of techniques and performance metrices along with limitations used by the scientists while providing the security in the network.

**Table 1:** Literature survey.

Author	Technique	Findings	Limitations
Ashok et al.^10^	Threats mitigation models	Assessed the performance of energy consumption, computational latency, scalability and deployment complexity measures	The possibility of dynamic threats while maintaining the records
Indumathi et al.^11^	IoMT and blockchain	Blockchain-based IoMT layered architecture which support the significant functions of patient centric concerns	The proposed mechanism leads to computational latency
Mehmood et al.^12^	Trust-based cooperative mechanism using cryptographic technique	The results are further demonstrated to increase the delivery ratio, reliability, and trust with reduced average delay	The communication delay and key storage
Biswas et al.^13^	Unified mechanism using blockchain	Implemented against various security measures such as access control, data storage, seamless migration	The blockchain data and long verification delay
Jara et al.^14^	Presented the architecture	Evaluated against impact of security, distance, round trip delay time etc	The architecture can be further enhanced into security method
Wang et al.^15^	Non-interactive privacy-preserving classification algorithm	Simulated against communication overhead and cost to measure the efficiency	Algorithms leads to storage and computational delay
Biswas et al.^16^	Conventional e-healthcare system using unified blockchain method	The data is accessed through off-chain restoring that is controlled via policy transactions and patient-centric transactions	Leads to communication overhead
Randall et al.^17^	Security and privacy mechanism	Combating COVID-19 using various networking and communication mechanisms	Cryptographic mechanism leads to delay and high cost for ensuring the security
Thapliyal et al.^18^	Robust authenticated key management scheme using blockchain	Proposed envisioned authentication and management of key	The key management leads to extra storage overhead in the system
Zachos et al.^19^	An accurate intrusion detection system	Identified various security attacks by developing various functional and non-functional testbeds	The computational overhead is much as compare to other schemes
Ekolle et al.^20^	A distributed solution for cybersecurity	Experimented the proposed scheme by comparing it with existing methods	Distributed solutions lead to management of heterogenous network issues
Dione et al.^21^	Reviewed IoT devices and blockchain mechanism	Proposed various future directions based on blockchain technology	Given a review paper

Ashok et al.^10^ have discussed various threats mitigation models which provide network-level, node-level, route-level and physical-level security. In addition, authors have discussed the assistance of evaluating several operating characteristics using specific use cases. The paper has assessed the performance of energy consumption, computational latency, scalability and deployment complexity measures. Further the metrics are compared among various security models to optimize the performance. Indumathi et al.^11^ have investigated the systematic investigation of current cloud storage, IoMT and blockchain by exploring their various necessities and challenges. In addition, the authors have developed and designed a novel blockchain-based IoMT layered architecture which support the vital functions of patient centric concerns. The authors have tested and validated the performance against various metrics in terms of audits.

Mehmood et al.^12^ have projected a trust-based communication mechanism for providing the privacy and reliability of wireless body area network. They have projected a trust-based cooperative mechanism using cryptographic technique. The proposed mechanism is simulated and validated using MATLAB simulator. The results are further demonstrated to increase the reliability, trust and delivery ratio with reduced average delay. The proposed mechanism further used a fuzzy-logic mechanism for benchmark the scheme. Biswas et al.^13^ have presented a unified mechanism for migrating the conventional and independent e-healthcare system using blockchain. The authors have addressed several data structure issues for blockchain and relational databases by describing the synchronization and conversion of large-scale e-healthcare information. The proposed mechanism is validated and implemented against various security measures such as access control, data storage, seamless migration etc. Jara et al.^14^ have presented the architecture and evaluated the capability to provide the continuous ubiquitous connectivity, monitoring, reliability, privacy and security. The authors have proposed an interconnection framework that is evaluated in AIRE project by focusing on patient breath. The proposed phenomenon is evaluated against impact of security, distance, round trip delay time etc. the validity of proposed mechanism is concluded against conventional framework. Wang et al.^15^ have designed a non-interactive privacy-preserving classification algorithm by encrypting the patient’s data using WBAN-gateway. The relay and privacy-preservation of proposed mechanism is maintained based upon their priorities. The detailed security analysis showed the proposed scheme achieving the relay and classification of information based upon their disease model. The proposed mechanism is simulated against communication overhead and cost to measure the efficiency. Biswas et al.^16^ have presented a solution for migrating the conventional e-healthcare system using unified blockchain method for accessing the medical information. The authors have used blockchain-based mechanism without requiring the modification of internal process. The information of patient is accessed through off-chain storage that is controlled through policy transactions and patient-centric transactions. Randall et al.^17^ goal is to protect the various security and privacy mechanism for combating COVID-19 using various networking and communication mechanisms. Further, Thapliyal et al.^18^ have designed a robust authenticated key management scheme using blockchain for smart healthcare systems. The authors have proposed envisioned authentication and management of key for analyzing the security and identifying the potential threats in the network. Zachos et al.^19^ have proposed an accurate intrusion detection system by proposing the hybrid anomaly-based system. The authors have identified various security attacks by developing various functional and non-functional testbeds. Ekolle et al.^20^ have introduced a distributed solution for cybersecurity by merging the individual security solutions into globalized profiles. The authors have experimented the proposed scheme by comparing it with existing methods. Dione et al.^21^ have reviewed IoT devices and blockchain mechanism in medical domain for showing their impact on medical records. The authors have proposed various future directions based on blockchain technology by ensuring privacy and security data.

Though various authors have proposed several cryptographic, blockchain-based and algorithms mechanism for maintaining the secrecy in the network. The existing schemes still suffers from various performance issues such as delay, reliability of maintain the records in the network, authenticity of devices, verification delay, storage overhead etc. It is further needed to propose a secure and transparent communication mechanism along with reduced delay, cost and storage process in the network.

## Proposed method

Trust is considered as one of significant and efficient mode of measuring the behavior and nature of communicating node in the network. Trust quantity is generally measured in two parameters such as 0 and 1 where 0 means no trust (the node is not trustworthy anymore in the network) and 1 means the node is highly trusted (recommended to be part of communication network). In comparison of other security and cryptographic algorithms and schemes, trust is defined as one of effective way of identifying the legitimacy of communicating node in the network. In this paper, the trust aggregation method will be used in order to measure the communicating behavior of a device.

### System model

**Figure 2:**
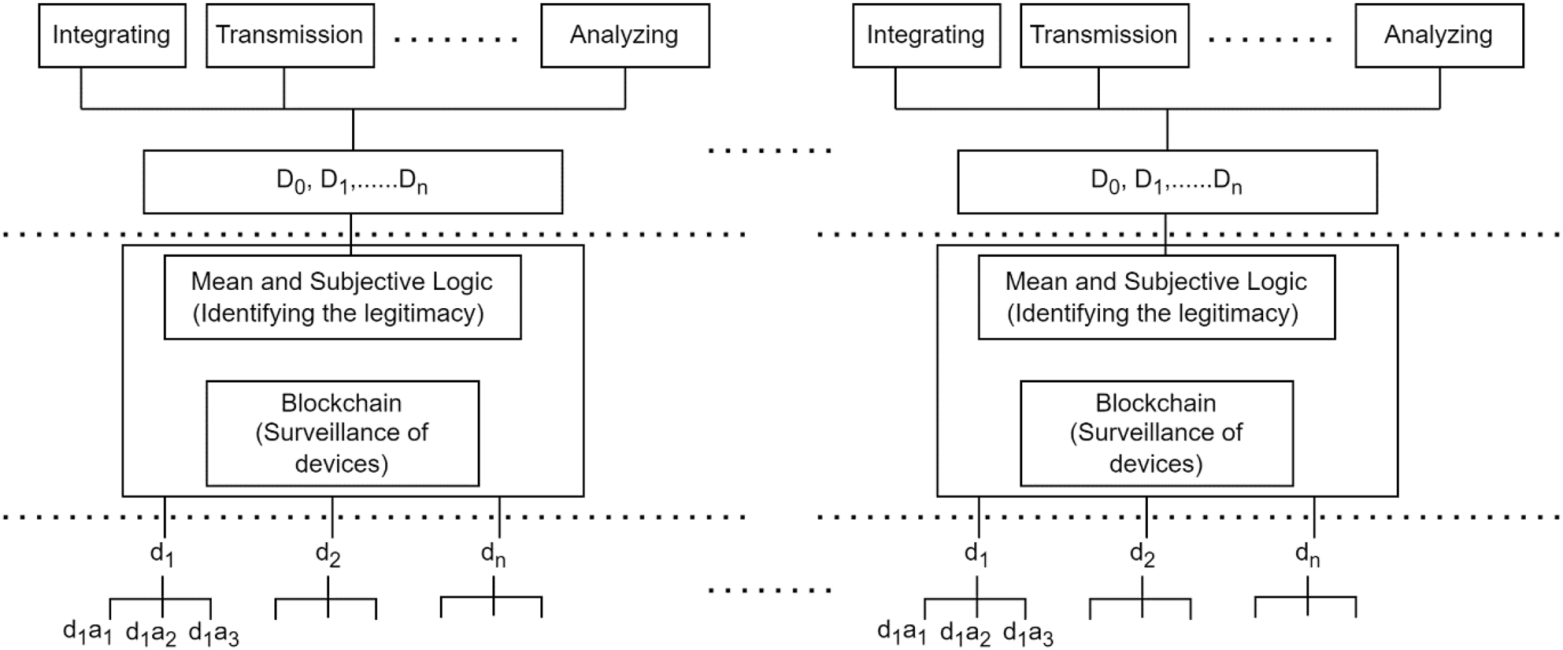
System model of proposed mechanism.

Figure 2 represents the system model of a network consists of ${D}_{c}$ number of communicating devices that are able to transmit and receive the information among each other. The ${D}_{1}, {D}_{2},\dots {D}_{n}$ are the number of devices that are considered and allowed to be a part of communication at a specific interval of time $T.$

In order to measure the trust or legitimacy of communicating device, number of metrics are considered to understand the working of trust computation using aggregate method. The nomenclature of the proposed mechanism is depicted in Table 2.

**Table 2:** Nomenclature of proposed model.

Abbreviation	Definition
I	Individuals
n	Number of information sources
Q	Quality
T	Trust
Dc	Communicating devices
$D1, D2,..Dn$	Number of communicated devices at particular time T
T	Time interval
ƞ	Nonce
Du	Unknown device

### Trust aggregation

Number of responses can be achieved of a single request from parallel number of paths. In order to understand trust aggregation in a simple way, let us take an example, where $X$ does not know $Z$, he asks him friends $(Ys)$ about $Z$. Different friends $(Ys)$ may have different ideas about that individual $Z$. Individual A should integrate the various ideas where he has received from $Ys$ about $Z$ to infer a unique idea about $Z$. Individual receive multiple recommendations and information from parallel sources where they can combine and finally decide the final judgement about an individual in order to simulate the behavior of that individual. The aggregation methods depend upon the trust’s models such as mean, fuzzy method and subjective logic combining approach. In this paper, we use the hybrid methods using mean and subjective logic aggregate methods to strengthen the trust computation process of a device.

### Mean and subjective logic combining operator aggregation method

It is defined as the summation of various information received from parallel sources in a particular amount of time $T$. If a device $D$ has $n$ various sources for an unknown device ${D}_{u}$, then device $D$ should aggregate the values obtained from others. If each device ${D}_{i}$ reports some believed value Bi and trust value ${TV}_{i}$ for device ${D}_{x}$, then the resultant aggregation of believe for ${D}_{x}$ will be computed as:

1 $${B}_{x}=\frac{\sum_{i=1}^{n}Tr\times Bi}{\sum_{i=1}^{n}TVi}.$$

Two operators are used to maintain parallel and serial opinion using consensus and reduction operators. In case, if P trust opinion to $Q$ has in context of information X is ${T}_{Q}^{P}=({m}_{Q}^{P}, {n}_{Q}^{P}, {\eta }_{Q}^{P} , {\chi }_{Q}^{P})$ and $Q{\prime}s$ opinion about information $X$ is ${T}_{X}^{P}=({m}_{X}^{P}, {n}_{X}^{P}, {\eta }_{X}^{P} , {\chi }_{X}^{P} )$ then to infer $P{\prime}s$ opinion, the $Q{\prime}s$ opinion about information $X$ can be reduced as follows:

2 $${T}_{X}^{PQ}={T}_{Q}^{P}\otimes {T}_{Q}^{P}=\left\{\begin{array}{c}{m}_{X}^{P:Q}={m}_{Q}^{P} {m}_{X}^{Q}\\ {n}_{X}^{P:Q}={m}_{Q}^{P} {n}_{X}^{Q}\\ {\eta }_{X}^{P:Q}={n}_{Q}^{P}+{\eta }_{X}^{Q}+{m}_{Q}^{P}{\eta }_{X}^{Q}\\ {\chi }_{X}^{P:Q}={\eta }_{X}^{Q}\end{array}.\right.$$

The subscript and superscript represent the trusted and trusting values. The reduction opinion is used to decrease and increase the beliefs/disbeliefs and uncertainty in transitive chains.

### Consensus operator

The consensus opinion represents the fair decision between two opinions $P and Q$ for the information $X$ with believes as ${T}_{X}^{P}=({m}_{X}^{P}, {n}_{X}^{P}, {\eta }_{X}^{P} , {\chi }_{X}^{P})$ and ${T}_{X}^{Q}=({m}_{X}^{Q}, {n}_{X}^{Q}, {\eta }_{X}^{Q} , {\chi }_{X}^{Q})$, their corresponding aggregation will be defined as:

3 $${T}_{X}^{P\phi Q}={T}_{Q}^{P}\theta {T}_{Q}^{P}=\left\{\begin{array}{c}{m}_{X}^{P\Phi Q}={m}_{X}^{P} {\eta }_{X}^{Q}+{m}_{X}^{P}\frac{{\eta }_{X}^{Q}}{{\eta }_{X}^{P}}+{\eta }_{X}^{P}-{\eta }_{X}^{P}{\eta }_{X}^{Q}\\ {m}_{X}^{P\Phi Q}={n}_{X}^{P} {\eta }_{X}^{Q}+{n}_{X}^{P}\frac{{m}_{X}^{Q}}{{n}_{X}^{P}}+{\eta }_{X}^{P}-{{\eta }_{X}^{P}\eta }_{X}^{Q}\\ {\eta }_{X}^{P\Phi Q}={\eta }_{X}^{P} {n}_{X}^{Q}+{n}_{X}^{P}\frac{{\eta }_{X}^{Q}}{{n}_{X}^{P}}+{\eta }_{X}^{P}-{{\eta }_{X}^{P}\eta }_{X}^{Q}\\ {\chi }_{X}^{P\Phi Q}={\chi }_{X}^{P}\end{array}\right..$$

The consensus operator effect is to reduce the uncertainty and improve the disbelief and belief.

4 $$\mathrm{Pr}\left({N}_{ri}\right)=\frac{{dt}_{i}}{\sum_{i=1}^{n}{dt}_{i}}.$$

The aggregated trust values computed between neighboring devices further strengthen the trust and legitimacy in the network. The devices’ having higher trust values are counted as most trusted and reliable that can be used to lead and coordinate the entire system.

The same method can be applied to determine the aggregation of trust value to a sink device obtained after getting the believes and trust value from its neighbors. If trust value from various neighbors to the destination node is computed as ${TrustDes}_{1}, {TrustDes}_{2}, \dots ..{TrustDes}_{n}$, and devices direct trust value defined as ${TV}_{1}, {TV}_{2}, \dots .{TV}_{n}$, the device trust $D$ to the destination as the aggregation result is computed as:

5 $${TrustDes}_{N}=\frac{\sum_{i\epsilon Ne(N)}{TV}_{i}\times {TrustDes}_{i}}{\sum_{i\epsilon Ne(N)}{TV}_{i}}.$$

**Algorithm 1:**
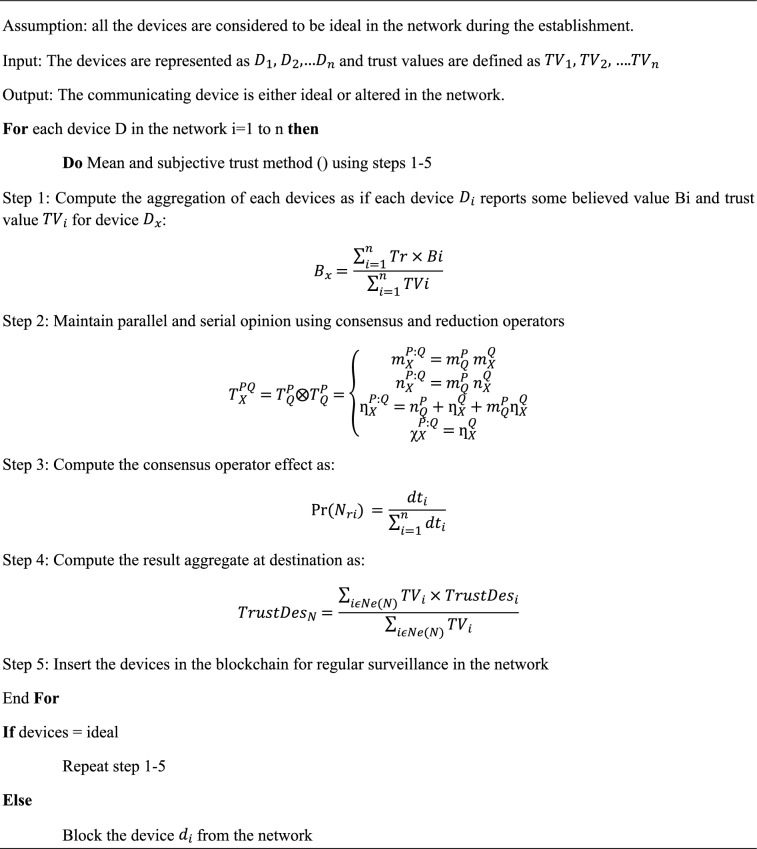
The trusted based blockchain enabled security mechanism for IoMT application.

The presented Algorithm 1 represents the computation and evaluation of communicating devices in the network based upon their behavior and trust values in the network. The trust of each device is computed using mean and subjective logic method. In addition, the computed trust devices are kept in blockchain for further surveillance. Repeat the step until the entire devices in the network and block the altered and untrusted devices from the network.

### Blockchain along with trust-based methods

As the devices are efficiently computed according to their behavior by identifying their trust values. The intruders may further insert software in order to later the ideal devices in the network that will drastically affect the overall performance of the network. Though trusted mechanisms are able to identify the legitimacy of each device, however, it is further needed to keep surveillance to the communicating devices where intruders may not compromise or alter the ideal devices. The identification of device alteration method by the intruder should be identified at the initial stage of the communicating process. In order to overcome the mentioned limitation, the trust-based method is further combined with blockchain technology that is transparent and may keep surveillance in the network. The blockchain creation and addition of devices mechanism is further described in the subsequent sections.

### Blockchain after aggregation schemes

The computed trust values of devices using aggregation methods are used integrated with the blockchain network in order to maintain the transparency and used to maintain the legitimacy and trust in the network. The devices having higher trust values are used to act as miners for further validating the incoming devices in the blockchain network.

### Blockchain creation

The blockchain of trusted devices is created in the network where the devices’ having higher trust values acts as miners for further validation and verification. The hex string is generated as,

6 $$Var TransactionHex=transaction.build().toHex().$$

### Addition of device in the blockchain

The devices having moderate trust values are keep on adding in the network after creating their hash values verified by the genesis device. The devices having lower trust values are keep on surveillance in the network where their reduction in trust values immediately gets removal from the blockchain network. The proposed mechanism is verified and validated against various performance metrices such as reliability, trust, delay and authentication.

## Performance analysis

**Figure 3:**
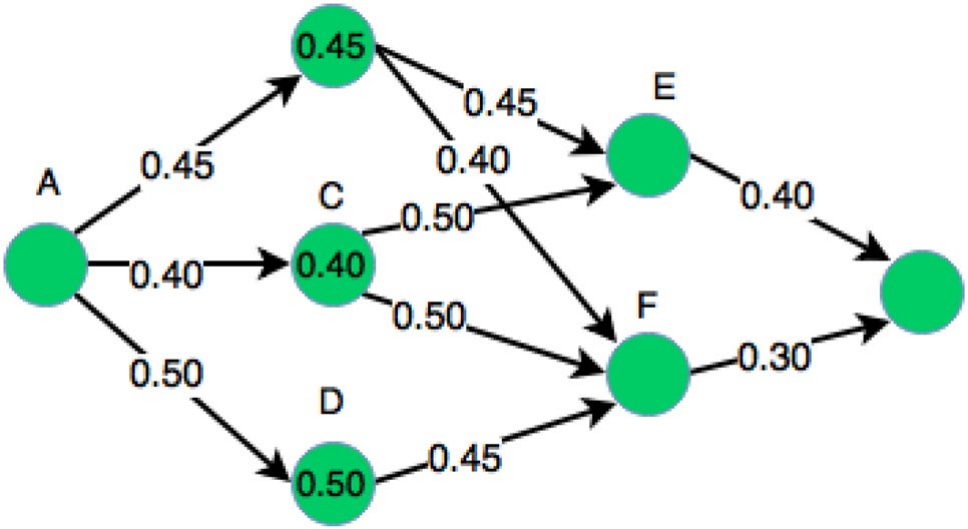
Trust values using mean and aggregate schemes.

The crisp trust graph is presented in Fig. 2 for simulating the aggregation method having various trust ranges [0–10] which are altered by a multiplication or division by 10. Table 3 represents the initial trust values that is present during the establishment of the network. The blank entry shows that devices are not communicated yet corresponding to each other. Once they started interacting among each other, the trust value keeps on changing and updating according to the aggregation method. Table 4 depicts the altered trust values after communication or transmission of information applying mean and subjective logic aggregation methods (Fig. 3).

**Table 3:** Mean and subjective logic results during establishment of network.

	A	B	C	D	E	F
A	–	0.7	0.8	0.7	0.7	0.8
B	–	–	0.8	0.9	–	–
C	0.9	0.8	–	0.8	0.9	0.9
D	0.8	0.9	0.9	–	0.8	0.8
E	0.9	–	–	0.8	–	0.9
F	–	0.8	0.8	–	–	–

**Table 4:** Mean and subjective logic results during transmission in network.

	A	B	C	D	E	F
A	0.9	0.6	0.7	0.7	0.6	0.9
B	0.8	0.8	0.7	0.6	0.4	0.8
C	0.8	0.5	0.6	0.9	0.7	0.8
D	0.7	0.4	0.8	0.8	0.7	0.9
E	0.9	0.8	0.6	0.6	0.6	0.6
F	0.8	0.7	0.7	0.7	0.7	0.7

### Baseline mechanisms

The proposed mechanisms are simulated and verified against two schemes such as cryptographic and blockchain-based technique. Wang et al.^15^ have designed a non-interactive classification algorithm that ensured privacy-preserving by encrypting the patient’s data using WBAN-gateway. The relay and privacy-preservation of proposed mechanism is maintained based upon their priorities. The detailed security analysis showed the proposed scheme achieving the relay and classification of information based upon their disease model. The projected mechanism is simulated against communication overhead and cost to measure the efficiency. In addition, Mehmood et al.^12^ have proposed a trust-based communication mechanism for ensuring the reliability and privacy of wireless body area network. They have proposed a trust-based cooperative mechanism using cryptographic technique. The proposed mechanism is simulated and validated using MATLAB simulator. The results are further demonstrated to increase the reliability, delivery ratio and trust with reduced average delay. The proposed framework is verified against these two methods for providing a transparent and secure communication approach.

## Results and discussion

The proposed mechanism is simulated against various security metrics over existing methods such as reliability, trust, delay and beliefs and disbeliefs.

### Reliability

The device D is said to be reliable if its trust value is above the maximum trust value range in the network. In case, if trust range of network is 0.45, the reliable device trust value would be above 0.45 to be remain existing in the network. The probability of reliability is measured over the trust range of current devices as ${dt}_{1}, {dt}_{2}, \dots .{dt}_{n}$ to its neighbors as ${N}_{1}, {N}_{2}, \dots ..{N}_{n}$ the selection probability of reliable device is computed as:

7 $${P}_{r}\left({N}_{ri}\right)=\frac{{dt}_{i}}{\sum_{i=1}^{n}{dt}_{i}}.$$

### Trust

The trust is measured as the ability to remain in the network for transmission of information $X$ among device $P and Q$. the trust values or range is computed as the integration of mean aggregation and subjective logic aggregation schemes to strengthen the trust computation process in the network.

### Delay

The delay is measured in respect of blockchain where the amount of time required the device or miner to validate the incoming device in the network. The devices’ having higher trust values verifies the devices that further determines the delay in the network to get verified by the miner.

### Beliefs and disbeliefs

It is defined as the count of believed and unbelieved devices in the network after applying trust schemes to identify the legitimacy of network. The more identified believed devices showed the legitimacy and stability in the network.

The following metrices can be further analyzed independently on confidentiality, integrity, authentication and availability security schemes of the network. However, the computed results can be summarized in terms of security services in the network. The integrity and disbelief of the network is compared with trust score where the devices having higher trust score would be highly legitimate and always be responsible for their computed information. In addition, the authentication and confidentiality security services can be measured with reliability where each device is reliable and not taking much amount to time to forward the incoming information from its neighbor. Further, delay and availability security service is again measured with trust score where the devices having higher trust values are ideal and never consume much resources in the network.

**Figure 4:**
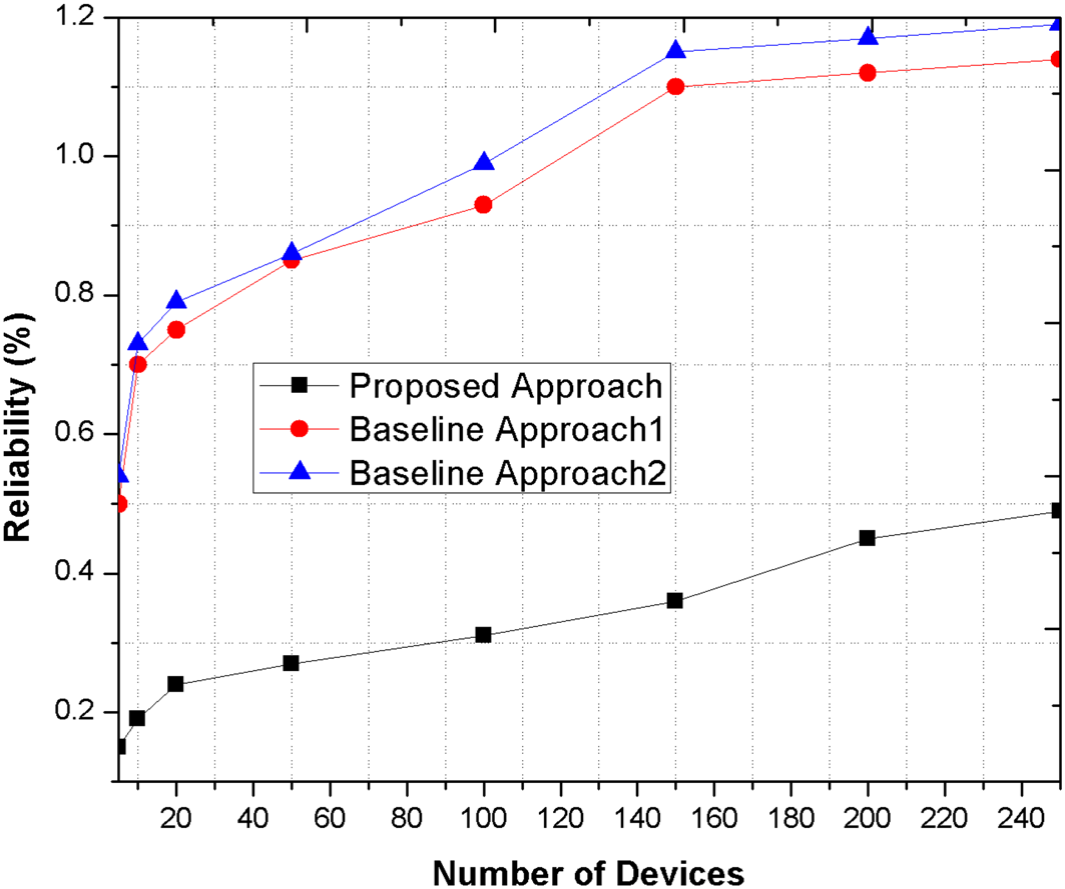
Comparison of the proposed method with other base line approaches for reliability metric.

The following parameters are measured against proposed and existing mechanisms as depicted in below graphs. Figure 4 shows the reliability of proposed and existing methods. The proposed mechanism is much reliable as compare to existing methods because of their integrated aggregation schemes that improves the trust and reliability among the devices while transmitting the information in the network.

**Figure 5:**
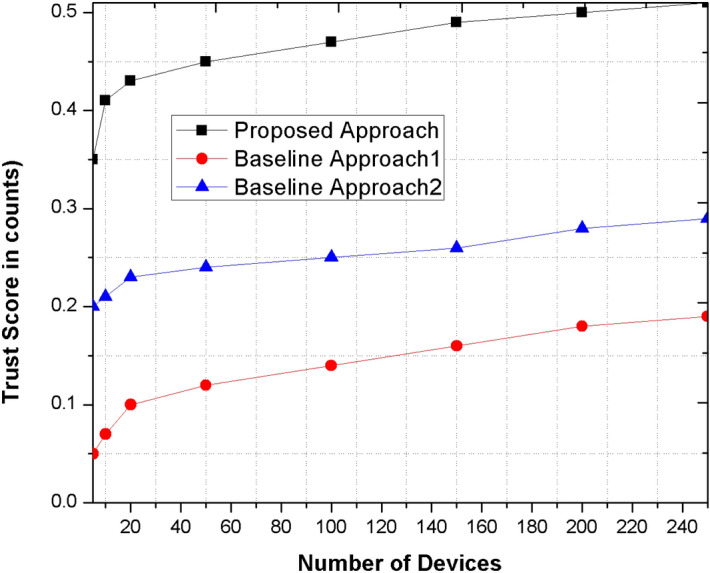
Comparison the proposed method with other base line approaches for trust score metric.

Figure 5 represents the trust score that are altered and changes during the communication among devices. The trust values of proposed mechanism are significantly higher as compare to existing method because of subjective logic combining method. The aggregated trust values computed between neighboring devices further strengthen the trust and legitimacy in the network. The devices’ having higher trust values are counted as most trusted and reliable that can be used to lead and coordinate the entire system.

**Figure 6:**
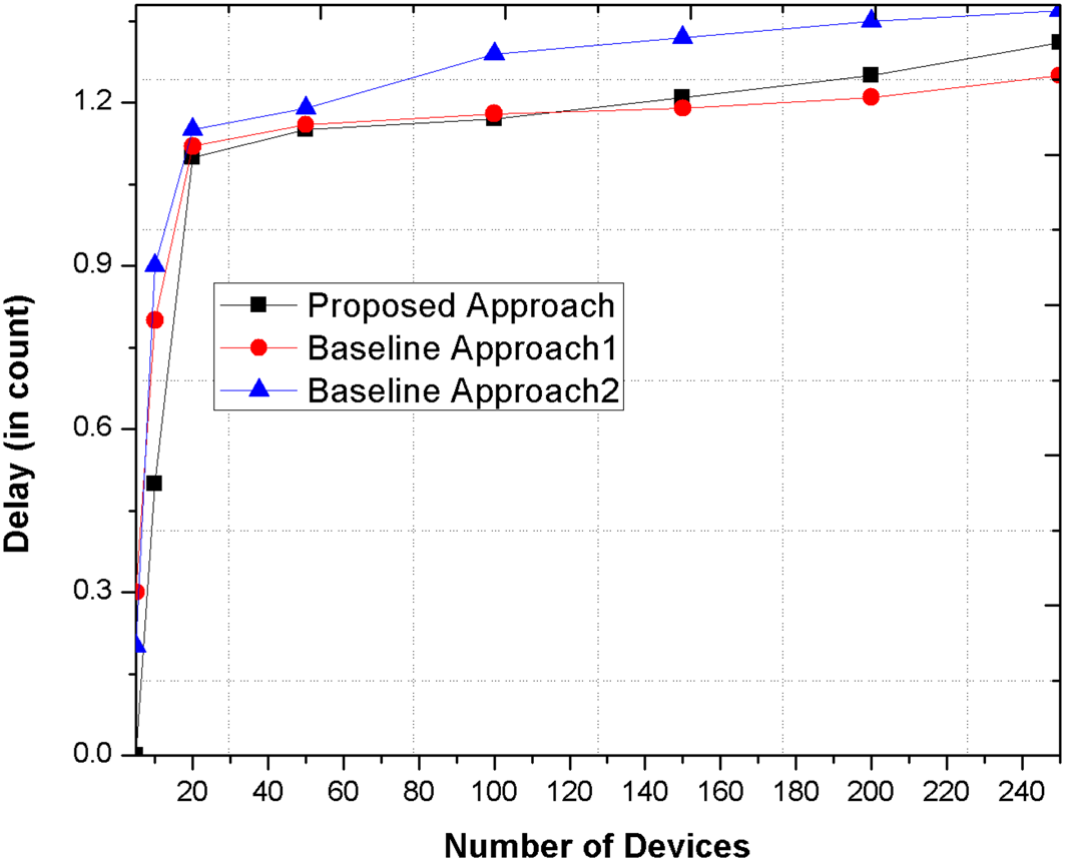
Comparison the proposed method with other base line approaches for delay metric.

Figure 6 depicts the delay means the number of devices to get verified in the network. The proposed mechanism uses blockchain in comparison of any cryptographic or probability-based method in the network. The proposed mechanism outperforms one existing mechanism because the delay using proposed method such as mean and subjective logic is efficient and stable in comparison of other methods as shown in the graph. However, in comparison of other existing mechanism such as Mehmood et al.^12^ that have proposed a trust-based communication method is faster as compare to proposed approach. The existing mechanism integrates the cryptographic and trusted schemes for validating and securing the devices. However, proposed approach integrates blockchain and trust-based mechanism that may slow down the process of validating, verifying and addition of block in the network. Therefore, the proposed approach has more delay in comparison of Mehmood et al.^12^ but with more security enhancements.

**Figure 7:**
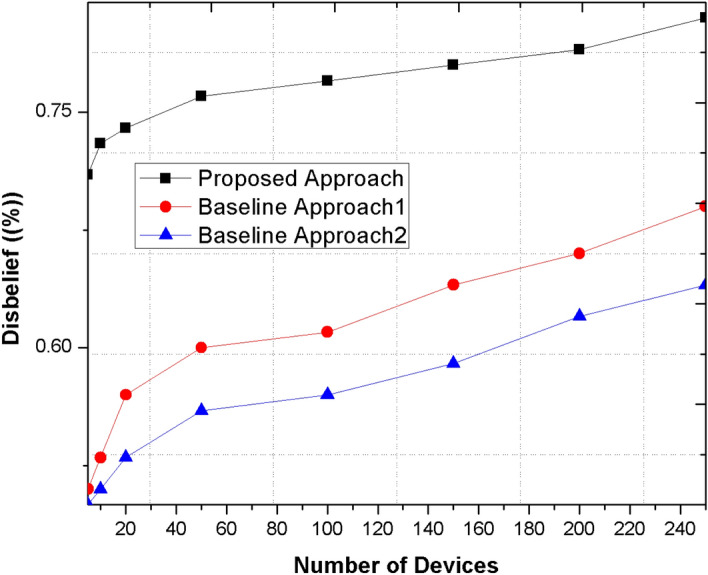
Comparison the proposed method with other base line approaches for disbelief metric.

Figure 7 represents the disbelieves in the network using proposed and existing methods. The believed devices of proposed mechanism is significantly higher than existing methods because of mean aggregation scheme that computes the trust value of each communicating device.

The above-mentioned parameters represent the outperformance of proposed mechanism in comparison of existing schemes.

The proposed mechanism is further analyzed over existing methods against cryptographic, trusted and blockchain technique as depicted in Table 5. The complexity of proposed approach is less as compare to both the existing schemes as encryption and decryption time increases the complexity, key management and storage overhead while transmitting the information in the network. Further the time and space complexity of various security schemes proposed by several authors is analyzed in Table 6.

**Table 5:** Comparison of existing methods trust methods with proposed framework along with their complexity.

Approaches	Technique	Cryptographic method	Trusted-based	Blockchain	Complexity
Baseline approach 1	Privacy-preserving by encrypting the patient’s data using WBAN-gateway	WBAN-gateway using privacy preserving encrypting method	–	–	${n}^{2}\mathrm{log}(n)$
Baseline approach 2	Trust-based communication mechanism	Cryptographic mechanism	Cooperative trusted scheme	–	${n}^{2}\mathrm{log}(n)$
Proposed approach	Trust-based method and blockchain	–	Mean and Subjective Trusted Method		$nlog(n)$

**Table 6:** Complexity analysis of various trusted schemes.

Trusted scheme	Space complexity	Time complexity	Overall complexity
Fuzzy logic recommendation system	${n}^{2}$	${n}^{2}$	${n}^{2}$
Trust-distrust mechanism	$n$	$n$	n × n
SMART trusted method	$\mathrm{log}n$	${n}^{2}$	${n}^{2}$ log n
Subjective logic and mean	$n$	$\mathrm{log}n$	n log n
Fuzzy evaluation matrix	$n$	$n$	n × n
TOPSIS cooperative trust-based scheme	$n$	${n}^{2}$	${n}^{2}$

## Conclusion

The e-health system not only reduces the gap between doctors and patients, but it also enables coordination and communication without the need to wait in long queues or be physically present in the hospital. The e-health system also improves the operation, diagnosis, and management of patient medical records in a more efficient and effective way. The proposed mechanism integrates trust-based and blockchain mechanisms to enhance the overall quality of the communication process and ensure efficient security. The proposed scheme outperforms existing mechanisms by utilizing combined aggregation methods such as mean and subjective logic, which improve the overall communication process while identifying the legitimacy of each device. Furthermore, the blockchain network enhances transparency and surveillance in the network. The proposed schemes have outperformed existing schemes in various security metrics.

Further, the improved security mechanism, along with reduced communication storage, cost, and increased efficiency, can be considered for future scope in this paper.
